# Total knee replacements using rotating hinge implants in polio patients: clinical and functional outcomes

**DOI:** 10.1007/s12306-022-00755-w

**Published:** 2022-08-10

**Authors:** V. Digennaro, M. Manzetti, B. D. Bulzacki Bogucki, F. Barile, A. Panciera, G. Viroli, R. Ferri, D. Cecchin, A. Ruffilli, C. Faldini

**Affiliations:** IRCCS Istituto Ortopedico Rizzoli, Via Giulio Cesare Pupilli 1, 40136 Bologna, Italy

**Keywords:** Poliomyelitis, Total knee replacement, Rotating hinge prosthesis, Knee society score, Arthroplasty, Outcome

## Abstract

Little evidences are available in the literature concerning the outcomes of total knee replacement (TKR) in poliomyelitis patients with severe knee deformities or degeneration. Encouraging results have been reported concerning the use of constrained implants, i.e., rotating hinge knee prosthesis (RHK), compared to less constrained ones. The purpose of this paper is to report our experience with rotating hinge total knee replacement, using only RHK prosthesis, to determine functional results, complications, and survival of TKR in poliomyelitis patients. We performed a retrospective chart review of 14 patients with a history of knee osteoarthritis following poliomyelitis that underwent primary TKR, for a total of 15 surgical procedure (one bilateral case). Preoperative and postoperative clinical measurements have been conducted for all patients using the Knee Society Score (KSS). Hip-knee angle, recurvatum knee angle, and Insall-Salvati index were evaluated with full weight-bearing panoramic view X-ray preoperatively and postoperatively. The 2-year postoperative clinical KSS significantly improved from the preoperative scores. The average clinical KSS improved from 32,9 (range 3–48) preoperatively to 77,4 (range 60–88) postoperatively (*P* value < 0.005). The average functional KSS improved from 32,5 (range 10–60) preoperatively to 59,4 (range 30–95) postoperatively (*P* value < 0.005). TKR is a successful treatment in improving knee function and patient’s quality of life. Using constrained implants, especially rotating hinge implants in polio patients with a quadriceps muscle weakness, could be a good alternative to maintain a physiological kinematics and reducing the revision rate due to knee instability.

## Introduction

Poliomyelitis is an infection caused by poliovirus, a member of the genus Enterovirus, belonging to the family of Picornaviridae [3]. Vertebrates, including humans, serve as natural hosts. Usually, the virus reaches the central nervous system (CNS) through the bloodstream. Here, it can cause extensive damage to the anterior horn cells of the spinal cord, causing limb paralysis. The virus may also spread to the posterior horn cells, motoneuron of the thalamus, and hypothalamus. In the case of brain stem involvement, also known as bulbar poliomyelitis, the symptoms are extremely severe and death from respiratory paralysis due to involvement of the respiratory muscles can occur.

The paralysis caused by the virus is flaccid, muscles are hypotonic, and even only a single muscle can be involved, due to the involvement of the motor unit at the level of the anterior horn of the spinal cord [11].

The typical clinical presentations in case of neurological involvement are two. The first one, post-polio syndrome (PPS), happens long after exposure to the virus. The second one, called acute anterior poliomyelitis (AAP), is more frequent when contracting the disease during adult life. Nevertheless, less than 1% of patients develop muscle paralysis, which affects more frequently the lower limbs.

In the literature, few studies have been designed to evaluate outcomes of total knee replacement (TKR) in patients with limbs affected by poliomyelitis [6, 10, 12, 13, 18]. In general, this procedure has been shown to promote excellent results in terms of pain reduction, functionality, and quality of life improvement. On the other hand, there are many concerns regarding the recurrence rate of knee instability, in particular regarding the hyperextension mechanism [12, 13] which is common among post-polio patients, and the rate of revision which is close to 7% and occurs on average after 6.2 years [15]. Overall outcomes are probably secondary to preoperative deformities, soft tissue laxity, and quadriceps deficiency; these are all factors to be taken into consideration before surgery.

The purpose of this paper is to report our experience with rotating hinge total knee replacement, using only RHK prosthesis, to determine functional results, complications, and survival of TKR in poliomyelitis patients.

## Materials and methods

We identified patients with a history of knee osteoarthritis in a polio-affected limb. All of them underwent TKR between October 2010 and May 2019 in a large tertiary referral center with great experience in complex arthroplasty. This retrospective study was approved by our institutional review board (protocol n° 0013141), and all the subjects have given their written informed consent to publish their clinical information and radiologic examinations.

A preoperative and postoperative clinical evaluation of each patient was performed using the clinical and functional *Knee Society Score (KSS)* [9]. An excellent outcome is defined when over 85, good between 70 and 84, fair between 60 and 69, and poor below 60. Using the Medical Research Council (MRC) scale, we were able to evaluate quadriceps strength and function in all patients.

The standard X-ray evaluation *consisted of* a full weight-bearing panoramic view, a latero-lateral, and an anteroposterior view. These allow an evaluation of the hip-knee angle (HKA) and recurvatum knee angle and eventual intra-articular knee deformities. Finally, we evaluated patellar height using the modified Insall-Salvati index [7].

In our department, the administration of the KSS questionnaire X-ray examinations is part of a standardized preoperative workup for all patients who will undergo a TKR. These evaluations are performed at 1 month of follow-up, 3 months, 6 months, 1 year, 2 years, and at the last follow-up. According to the Knee Society radiographic evaluation and scoring method system [4], any sign of aseptic loosening or radiolucency was reported. Additionally in this series, we performed a pre-operative CT scan of the knee to better determine the size of the tibial plateau and the diameter of tibial and femoral canals, since one of the most common polio-related deformities is a very narrow diaphyseal canal [6, 13]. We chose a rotating hinge implant for all patients, because it provides more knee stability, reducing the rate of instability and the rate of revision procedures [18]. Nine Smith + Nephew™ RT rotating hinge (*Trademark of Smith* + *Nephew™*) and six Endomodel Link™ rotating hinge (*Trademark of Waldemar Link GmbH & Co™*) *were implanted* in this series. According to the indications provided by the manufacturing company, the Smith + Nephew RT have been implanted in patients with larger tibial plateau; instead, the Endomodel Link were implanted in patients with a smaller tibial plateau. *In fact, the smallest size of the Smith* + *Nephew RT tibial plateau has a medial–lateral length of 60 mm, which could not be adapted to the small tibial plateau and is way larger than the smallest tibial plateau of Endomodel link***.** These implants were chosen due to their high degree of adaptability and the surgeon’s familiarity with the instrument set. All surgical procedures were performed by the same surgeon, an arthroplasty fellowship-trained individual.

**Fig. 1:**
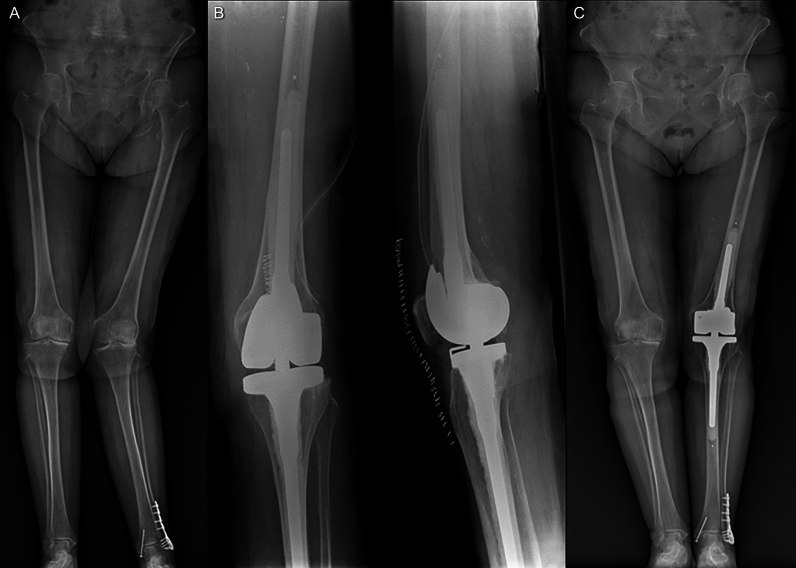
Case T.C., 84-year-old female; **A** The preoperative full weight-bearing X-ray shows left knee arthritis, more severe on the lateral side, with a severe valgus deformity. **B** Two projection knee X-ray shows the postoperative RX evaluation of the hinged implant. **C** *The 1-year postoperative X-ray shows a good correction of the initial deformity*

**Fig. 2:**
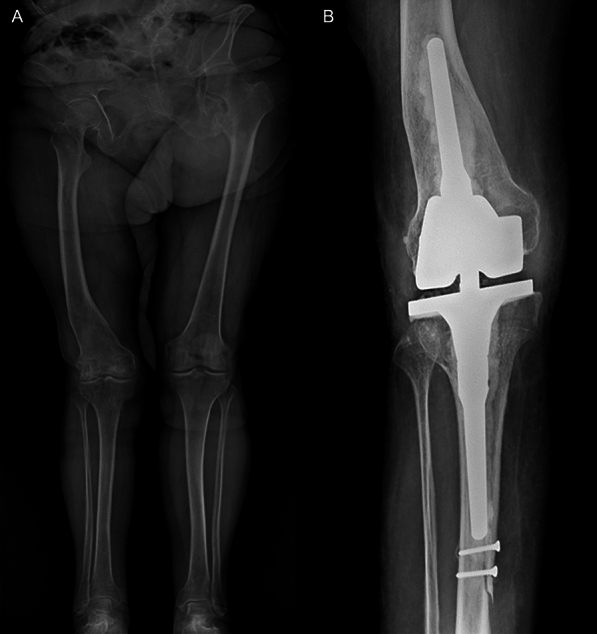
Case B.L., 77-year-old female; **A** The preoperative full weight-bearing X-ray shows right knee osteoarthritis with a femoral diaphysis deformity. **B** 1-year postoperative anteroposterior right knee X-ray shows the good positioning of the implant. During the reaming of the tibial diaphyseal canal, very narrow, an incomplete tibial shaft fracture was reported and synthesized with two mono-cortical screws

**Fig. 3:**
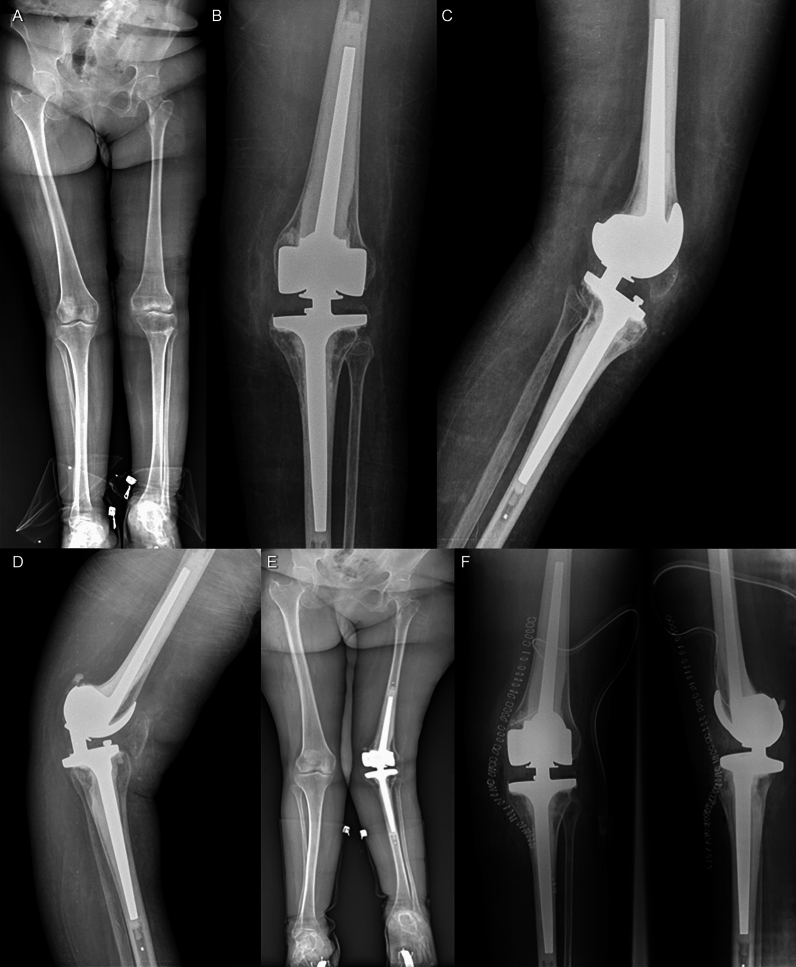
Case N.G.E. 66-year-old female; **A** The preoperative full weight-bearing X-ray shows a radiographic aspect of left knee osteoarthritis marked on the medial side. **B–C** 1-month postoperative left knee anteroposterior and latero-lateral X-ray showing the good positioning of the implant. **D** 1-year postoperative left knee anteroposterior X-ray showing the recurrence of hyperextension. **E** 1-year postoperative full weight-bearing X-ray shows the postoperative left knee valgus deformity. **F** Postoperative anteroposterior and latero-lateral left knee X-ray shows the final result after the substitution of the bush and the polyethylene insert

All patients were positioned lying supine and the knee in a stable 90° flexion. The standard anteromedial parapatellar approach was used in all patients. A collateral ligaments release was performed during all procedures; this allows to achieve fewer stress forces on the rotating hinge mechanism of the implants, to facilitate patellar eversion and obtain a smooth tracking over the center of the knee intraoperatively [20]. Any bone gap was managed with the use of femoral or tibial wedges. In every case, we used cemented implants, allowing for great primary stability, which is very important, especially in patients with neurological deficiencies. Figures 1, 2, and 3 illustrate three cases of our population.

### Statistical analysis

The Shapiro–Wilk test was used to verify normal distribution of the clinical and functional KSS. *Statistical analysis was performed through the T-Test for dependent variables by comparing preoperative clinical and functional KSS with postoperative clinical and functional KSS.* Statistical significance was defined by a *p*-value < 0.005. Data analysis was performed using the R software (© The R Foundation. Released 1993).

## Results

During the study period, we selected a total of 14 patients, in which 15 surgical procedures were performed (one bilateral case). An average follow-up of 3,1 years (range 2–10 years) was conducted. Our cohort presented a female prevalence (F/M 11:4), with an average age at the time of surgery of 63,9 years (range 41–84). The preoperative data are summarized in Table 1.

**Table 1:** Preoperative data concerning KSS, quadriceps functionality, and radiographic parameters

Patient	Age	Sex	Clinical KSS	Functional KSS	MRC scale	HKA angle	Recurvatum knee angle	Patellar ratio	Follow-up
1	55	F	33	42	2\5	10° valgus	0°	0,6	10 yrs
2	63	F	47	20	1\5	5° varus	23°	0,52	3 yrs
3	68	F	32	33	1\5	15° valgus	12°	0,61	3 yrs
4	63	M	42	15	1\5	6° valgus	22°	0,72	3 yrs
5	73	F	48	25	1\5	6° valgus	15°	0,6	2 yr
6	84	F	10	25	1\5	22° valgus	–	0,87	5 yr
7	84	F	36	40	2\5	7° varus	10°	0,78	4 yr
8	41	M	3	10	0\5	22° valgus	20°	0,64	2 yrs
9	62	F	28	60	4\5	3° valgus	–	0,52	2 yrs
10	56	F	24	10	0/5	24° valgus	20°	0,85	2 yr
11	73	F	28	25	1\5	3° varus	10°	0,72	2 yrs
12	44	M	36	43	2\5	12° valgus	12°	0,75	2 yrs
13	51	M	42	45	3\5	20° valgus	–	0,93	3 yrs
14	63	F	39	45	3\5	8° valgus	–	0,62	2 yrs
15	79	F	46	50	3\5	9° varus	–	0,59	2 yrs

Concerning clinical results, we chose to evaluate the outcomes at 2 years of follow up, since only 2-year results were reported in all patients. The postoperative clinical KSS was in most cases excellent or good, significantly improving from the preoperative scores.

The average clinical score improved from 32,9 (range 3–48) preoperatively to 77,4 (range 60–88) postoperatively at 2-year follow-up (*P* value < 0.005). The average function score improved from 32,5 (range 10–60) preoperatively to 59,4 (range 30–95) postoperatively at 2-year follow-up (*P* value < 0.005).

According to the *T*-test for dependent variables, there was a statistical difference between the preoperative and 2-year follow-up measurements on clinical scores. According to the MRC scale, every patient had a reduced quadriceps strength. An excellent stability was achieved in all patients except two who presented mechanic failure that required the revision of the polyethylene insert and the bush one year after the first procedure and another one who reported the recurrence of knee recurvatum one year after surgery. Only this knee needed the use of brace.

**Fig. 4:**
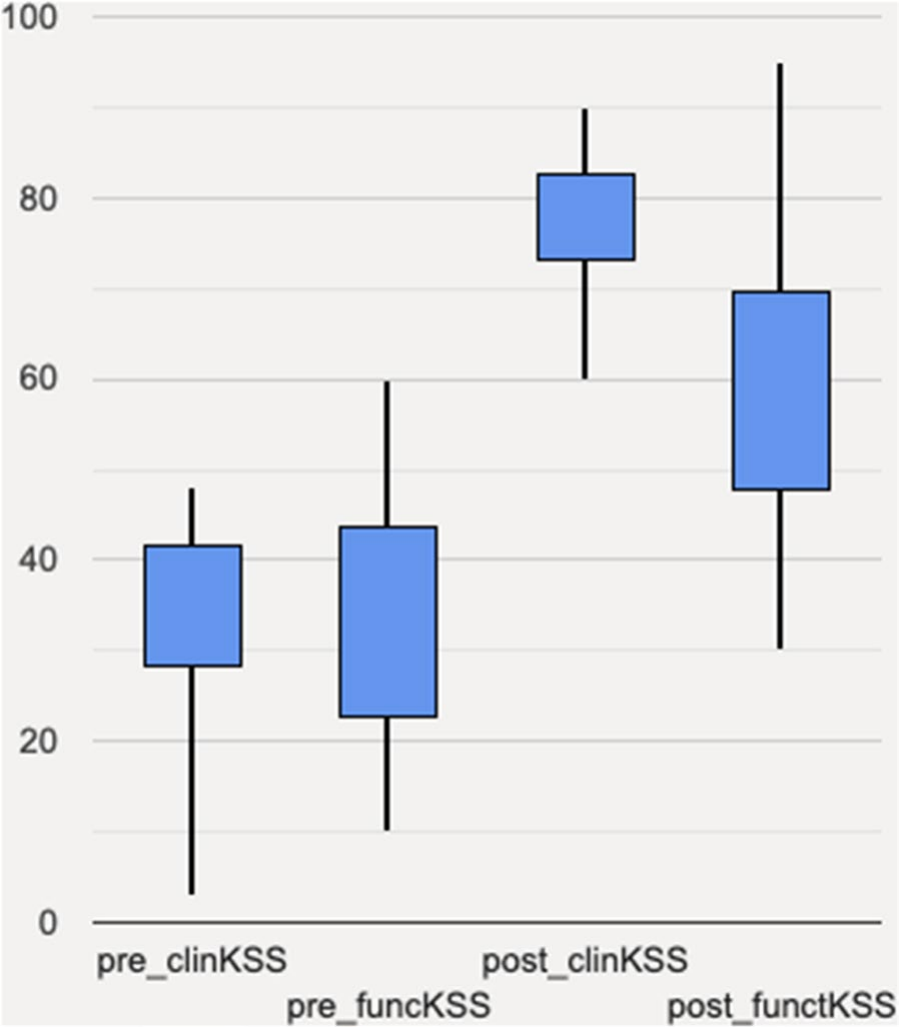
Clinical scores boxplot shows how the values improve and distribute in our population

One patient had an intraoperative tibial shaft fracture, who required fixation with two cortical screws and no-weight-bearing on the right knee for six weeks. At last follow-up, X-ray showed complete bone healing. No patient developed arthrofibrosis after TKR, and no progressive radiolucent lines were reported in our series. Table 2 and Fig. 4 summarize all the findings and compare preoperative and 2-year follow-up measures (Table 3).

**Table 2:** 2-year follow-up clinical and radiographic data

Patient	Age	Implant type	Clinical KSS	Functional KSS	H–K-A angle	Recurvatum knee angle	Patellar ratio	Complications
1	55	RT rotating hinge S&N	78	75	4° valgus	–	0,8	–
2	63	Endomodel Link rotating hinge	88	45	3° varus	7°	0,7	Mechanic failure
3	68	RT rotating hinge S&N	80	55	3° valgus	3°	0,76	–
4	63	RT rotating hinge S&N	83	40	2° valgus	9°	0,88	Recurrence of knee recurvatum
5	73	RT rotating hinge S&N	90	45	2° valgus	4°	0,89	–
6	84	RT rotating hinge S&N (Left side)	75	50	4° valgus	–	0,84	–
7	84	RT rotating hinge S&N (Right side)	80	65	3° varus	5°	0,94	–
8	41	Endomodel Link rotating hinge	69	70	5° valgus	8°	0,76	–
9	62	Endomodel Link rotating hinge	76	95	3° valgus	–	0,87	–
10	56	Endomodel Link rotating hinge	60	30	2° valgus	6°	0,89	–
11	73	RT rotating hinge S&N	70	50	3° varus	3°	0,85	–
12	44	Endomodel Link rotating hinge	75	75	4° valgus	4°	0,80	–
13	51	RT rotating hinge S&N	83	65	2° valgus	–	0,87	–
14	63	RT rotating hinge S&N	83	62	3° valgus	–	0,78	–
15	79	Endomodel Link rotating hinge	71	70	3° varus	–	0,75	–

**Table 3:** Mean preoperative and 2-year follow-up scores

	Preoperative average	2-year follow-up average	*p*-Value
Clinical KSS	32,9	77,4	< 0.001
Functional KSS	32,5	59,4	< 0.001

## Discussion

According to our study, total knee replacement represents a successful treatment with improvement of knee function and patient’s quality of life. Polio patients tend to develop abnormal alignment of the lower limb and muscle hypotonia, especially of the quadriceps muscle, with an associated ligamentous laxity that predisposes to knee pathologies at a young age [15]. The most common clinical features found in polio patients are [13]: angular deformities of the metaphysis, tibial external rotation, excessive valgus, bone loss, narrow femoral and tibial canal, reduced power of the femoral quadriceps muscle, ligamentous laxity, and finally genu recurvatum or hyperextension.

Clearly, this biomechanical situation leads to problems such as osteoarthritis, pain, arthrosis, ligamentous laxity, and painful extension that can be corrected at first with osteotomies and soft tissue releases, but almost inevitably will require total prosthetic replacement of the affected knee [15].

For these reasons, total knee replacement in these patients represents a challenging procedure. When approaching knee osteoarthritis in a polio patient, it is important to note that the patient tries to keep his knees fully extended during the loading phase of the gait [18]. In fact, the quadriceps deficit is balanced by bringing the gravity line in front of the knee joint axis, therefore using the patient’s own weight and the knee posterior capsule as a sort of locking mechanism. This biomechanical condition can be achieved through two compensation mechanisms [5, 16, 21]. The first mechanism is rarely present in the polio population and consists of leaning the pelvis and the trunk forward. This occurs in the presence of a flexion contracture of the knee due to paralysis of the quadriceps combined with retraction of the hamstring’s muscles. For this compensation to be effective, the function of the pelvic-trochanteric muscles (i.e., the piriformis, the internal and the external obturator) and the foot joints must be preserved to allow good contact of the foot with the ground [18]. Another mechanism is to block the knee in hyperextension, or genu recurvatum, or an exaggeration of the passive standing position. Hyperextension is a mechanism on which polio patient commonly rely for stability and walking. Correcting this deformity during the surgical procedure, can have a negative impact on the patient’s gait. However, and excessive postoperative knee hyperextension increases the rate of recurrence of knee instability [12, 13].

Few studies have been dedicated to total knee replacement in polio patients, with encouraging functional outcome. Patterson et al. [13] described a good pain relief and stability achievement in 9 polio patients who underwent TKR. However, there was a 25% of recurrence of recurvatum knee, which was uncommon with constrained implants compared with other less constrained [13]. Giori et al. [6] also reported a good improvement of KSS and pain relief in patients with poliomyelitis with a greater that antigravitary quadriceps strength. In those with more severely affected knees, recurrent instability was common. Thus, in current practice, Giori et al. [6] suggested using highly constrained implants in patients with less than antigravitary or absent quadriceps strength.

Tigani et al. [18] reported encouraging results concerning the use of a customized rotating hinge implant design with about 5° of hyperextension built in. In fact, the authors observed excellent articular stability in all patients except one, during a follow-up ranging from 2 years to 8.5. There is still a debate about the use of constrained implants in polio patients. In more severely affected patients with poor muscle power, providing a more intrinsic stability is useful to avoid the recurrence of hyperextension. Nevertheless, a more intrinsic stability also increases the stress transfer to the fixation interface and subsequently increases the risk of loosening. The first generation of rotating hinge was unsuccessful for this reason [1, 19].

The modern rotating hinge prostheses we used mimic a more physiological kinematics, giving a better distribution of shearing forces [18]. In fact, long-term results of these prostheses are encouraging. The survivorship rate reported in the literature swings from 75.8% to 96.1% at 15 years of follow-up [2, 14] in primary TKR setting, and from 65.1% to 95% in revision surgery [8, 17].

We recognize that our study has limitations. First, the limited cohort even if that nowadays patients with poliomyelitis affected limbs are exceedingly rare. Moreover, this pathology affected multiple joints in some of our patients, restricting their mobility and quality of life. That is why it is difficult to give a meaning to our findings. On the other hand, we obtained a significative improvement of clinical scores and of quality of life, with a very low rate of complications, using rotating hinge implants for all patients. In addition, good clinical and functional results were obtained in two patients with 0/5 MRC scale quadriceps strength value. These are cases that contradict the current practice reported by Giori et al. [6] where, however, various types of prostheses were used instead of a rotating hinge implants for all patients.

## Conclusion

According to our study report, total knee replacement in polio patients is a successful treatment in improving knee function and patient’s quality of life; however, the revision rate is high. Using a more constrained implant, especially a rotating hinge implant in those patients with a quadriceps muscle weakness, could be a good alternative to maintain a physiological kinematics and reducing the revision rate due to knee instability. Due to severe abnormalities following polio, we suggest to perform this procedure only in dedicated center with high volume of complex TKR.

## References

[1] Barrack RL (2001) Evolution of the rotating hinge for complex total knee arthroplasty. Clin Orthop Relat Res. 10.1097/00003086-200111000-00038 11716398 10.1097/00003086-200111000-00038

[2] Bistolfi A, Lustig S, Rosso F, Dalmasso P, Crova M, Massazza G (2013) Results with 98 Endo-Modell rotating hinge prostheses for primary knee arthroplasty. Orthopedics 36:e746–e752 23746036 10.3928/01477447-20130523-19

[3] Bodian D, Horstmann D (1965) Polio viruses. In: Horsfall FL JI (ed) Viral Ricketts infect man Lippincott Williams & Wilkins, Philadelphia, pp 430–473

[4] Caplan N, Kader DF (2014) The knee society total knee arthroplasty roentgenographic evaluation and scoring system. Class Pap Orthop Springer-Verlag, London Ltd, pp 193–195

[5] Costanzo DPN (1955) Problemi terapeutici nel ginocchio paralitico e poliomielite. Ortop e Traumatol dell’apparato locomotore, pp 139–178

[6] Giori NJ, Lewallen DG (2002) Total knee arthroplasty in limbs affected by poliomyelitis. J Bone Jt Surg Ser 84:1157–1161 10.2106/00004623-200207000-00010 12107315

[7] Grelsamer RP, Meadows S (1992) The modified Insall-Salvati ratio for assessment of patellar height. Clin Orthop Relat Res 282:170–176 1516309

[8] Gudnason A, Milbrink J, Hailer NP (2011) Implant survival and outcome after rotating-hinge total knee revision arthroplasty: a minimum 6-year follow-up. Arch Orthop Trauma Surg Springer 131:1601–1607 10.1007/s00402-011-1330-5 21656196

[9] Insall JN, Dorr LD, Scott RD, Scott WN (1989) Rationale of The Knee society clinical rating system. Clin Orthop Relat Res. 10.1097/00003086-198911000-00004 2805470

[10] Jordan L, Kligman M, Sculco TP (2007) Total knee arthroplasty in patients with poliomyelitis. J Arthroplast Churchill Livingstone 22:543–548 10.1016/j.arth.2006.03.013 17562411

[11] Mehndiratta MM, Mehndiratta P, Pande R (2014) Poliomyelitis: historical facts, epidemiology, and current challenges in eradication. Neurohosp. 10.1177/1941874414533352 10.1177/1941874414533352 PMC4212416 25360208

[12] Moran MC (1996) Functional loss after total knee arthroplasty for poliomyelitis. Clin Orthop Relat Res. 10.1097/00003086-199602000-00033 8625587 10.1097/00003086-199602000-00033

[13] Patterson BM, Insall JN (1992) Surgical management of gonarthrosis in patients with poliomyelitis. J Arthroplast Churchill Livingstone 7:419–426 10.1016/s0883-5403(07)80034-9 1431926

[14] Petrou G, Petrou H, Tilkeridis C, Stavrakis T, Kapetsis T, Kremmidas N, Gavras M (2004) Medium-term results with a primary cemented rotating-hinge total knee replacement. A 7- to 15-year follow-up. J Bone Jt Surg Ser B. 10.1302/0301-620X.86B6.14708 10.1302/0301-620x.86b6.14708 15330020

[15] Prasad A, Donovan R, Ramachandran M, Dawson-Bowling S, Millington S, Bhumbra R, Achan P, Hanna SA (2018) Outcome of total knee arthroplasty in patients with poliomyelitis: a systematic review. EFORT Open Rev. 10.1302/2058-5241.3.170028 30034816 10.1302/2058-5241.3.170028 PMC6026880

[16] Vittorio Putti (1922) Rapporti statici fra piede e ginocchio nell’arto paralitico. Chir Organi Mov 6:125–38

[17] Sanguineti F, Mangano T, Formica M, Franchin F (2014) Total knee arthroplasty with rotating-hinge Endo-Model prosthesis: clinical results in complex primary and revision surgery. Arch Orthop Trauma Surg 134(11):1601–1607. 10.1007/s00402-014-2061-1 10.1007/s00402-014-2061-1 25179893

[18] Tigani D, Fosco M, Amendola L, Boriani L (2009) Total knee arthroplasty in patients with poliomyelitis. Knee Knee 16:501–506 19443223 10.1016/j.knee.2009.04.004

[19] Wang CJ, Wang HE (2000) Early catastrophic failure of rotating hinge total knee prosthesis. J Arthroplast. 15:387–391 10.1016/s0883-5403(00)90877-5 10794238

[20] Yang JH, Yoon JR, Oh CH, Kim TS (2012) Primary total knee arthroplasty using rotating-hinge prosthesis in severely affected knees. Knee Surg Sport Traumatol Arthrosc 20:517–523 10.1007/s00167-011-1590-1 21773833

[21] Zanoli R, Franz A (1961) Il ginocchio poliomielitico. In L C (ed) Ter Ortop e Chir della Polio Bologna, pp 131–144

